# Curvature analysis of CI electrode arrays: a novel approach to categorize perimodiolar positions without anatomical landmarks

**DOI:** 10.1007/s00405-024-08917-1

**Published:** 2024-08-30

**Authors:** Alexander Mewes, Jan Dambon, Goetz Brademann, Matthias Hey

**Affiliations:** 1 Christian-Albrecht University of Kiel, Kiel, Germany; 2 Department of Otorhinolaryngology, Head and Neck Surgery, Universitätsklinikum Schleswig-Holstein (UKSH), Campus Kiel, Kiel, Germany

**Keywords:** Cochlear implant, Curvature, Over-insertion, Under-insertion, Angular depth of insertion, aDOI

## Abstract

**Purpose:**

Perimodiolar electrode arrays may be positioned regular, over-inserted or under-inserted into the cochlea depending on the cochlear size and shape. The study aimed to examine whether there are differences between these groups in the local curvature along the intracochlear array. Individual curvature variables were developed to categorize the groups and the relationship between the curvature and the angular insertion depth at the electrode tip was analyzed.

**Methods:**

The curvature along the intracochlear array was measured in the CBCT image of 85 perimodiolar electrodes of a single type. The mean curvature and the ratio of the mean curvature at contacts E14–16 to the mean curvature at E7–8 (bowing ratio) were calculated across the array, and its true positive rate (TPR) and false positive rate (FPR) were calculated to establish optimal threshold values to categorize the groups.

**Results:**

68.2% of the cases were categorized as regular positioned, 22.4% had an over-insertion and 9.4% had an under-insertion. The mean curvature was significantly weaker with under-insertion (< 342°) than with normal insertion depth (≥ 342°). With an over-insertion, the bowing ratio was < 1 and otherwise > 1. Both the mean curvature and bowing ratio were found to have an optimal threshold value with high TPR (= 1.00) and low FPR (≤ 0.06) for categorizing under-insertion and over-insertion, respectively.

**Conclusion:**

Curvature analysis is a useful tool to assess if a perimodiolar electrode array has been inserted deep enough into the cochlea. Independent of critical anatomical landmarks, over-inserted arrays and under-inserted arrays could be well categorized by using individual curvature variables. The results need to be validated using additional data sets.

## Introduction

**Fig. 1:**
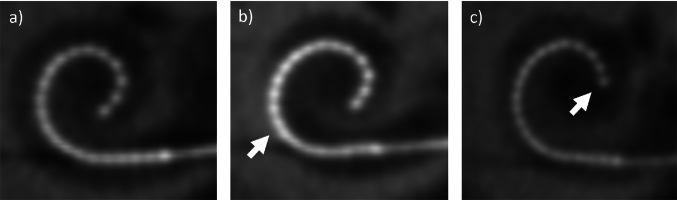
Cochlear view from CBCT images showing left to right examples of a regular electrode position (**a**), over-insertion (**b**) and under-inserted array (**c**). The white arrow in (**b**) indicates medio-basal contacts at which the electrode array bends towards the lateral wall. In (**c**), the white arrow illustrates that the array has not been inserted a full turn (360°) into the cochlea

The positioning of the CI electrode array as close as possible to the cochlear spiral ganglion neurons is of key importance for the electrode-neuron interface [1–5]. Perimodiolar electrode arrays have been designed to achieve a close position to the modiolar wall by their mechanical properties in conjunction with an adapted insertion technique. In comparison to straight electrode arrays, perimodiolar arrays yield lower stimulation thresholds, a more focused stimulation, and larger electrophysiological responses of the auditory nerve [6–10]. The perimodiolar position can vary with different array types [4] and with the same perimodiolar electrode array type [11]. Sub-optimal electrode array placements such as over-insertion or under-insertion may occur when adhering to surgical guidelines [12, 13]. An over-insertion is indicated by some electrode contacts being closer to the lateral wall of the cochlea than to the modiolar wall [11, 14–16], and an under-inserted array is associated with a shallow insertion depth of the electrode tip [16] (Fig. 1). Variability in cochlear size and shape have been observed to be contributing factors to sub-optimal positions of the electrode array [12, 17]. Sub-optimal positions may impair the electrode-auditory nerve interface by increasing the distance of the electrode contacts to the spiral ganglion neurons. To describe the quality of the position of a slim modiolar electrode array, Aschendorff et al. categorized 45 of these perimodiolar arrays into “good”, “moderate”, and “poor” by subjectively rating coronal cone beam CT (CBCT) images [11]. Such an evaluation method seems to be feasible for integration in the clinical routine because of its simplicity. Its limitations are that lacks standardization and quantification, and thus does not allow comparisons between different raters. To assess the electrode position quantitatively, localization parameters such as the wrapping factor (or medial–lateral position) [11, 18], electrode-modiolus distances (EMD) [3, 4, 19–22], and the angular insertion depth (aDOI) [4, 11, 22–24] have become clinically established. These parameters characterize the electrode position in a generalized way only (wrapping factor, medial–lateral position) or they depend on the localization of critical landmarks like the round window and the mid-modiolar axis in the CT image (EMD, aDOI, medial–lateral position). For the detection of the mid-modiolar axis there is especially a large variability between different raters but also between different techniques in assessing the CI electrode position [22, 25, 26]. In comparison to these critical landmarks, the detection of electrode contacts is robust from our clinical practice.

Here we present a method to assess the quality of perimodiolar electrode positions from CBCT images using solely an analysis of the local curvature along the intracochlear array. Analyzing the curvature along the electrode array is a simple tool that only requires a radiographic image to be coplanar with the basal turn of the cochlea (cochlear view) and in which the intracochlear electrode contacts can be detected. We postulate that this method can identify differences in the electrode position with respect to the presence of an under-insertion or over-insertion in a single perimodiolar electrode type.

The aim of this study was to examine whether there are differences in the curvature along the electrode array between a regular electrode position, an under-insertion and over-insertion of the array. Individual curvature variables should be developed to categorize under-inserted and over-inserted electrode arrays. In addition, it should be analyzed if there is a relationship between the curvature and the angular depth of insertion of CI electrode arrays.

## Methods

### Design and setting of the study

This cross-sectional study investigated the diagnostic accuracy in assessing the quality of the electrode array position using an analysis of the local array curvature. All measurements were conducted on a training data set of post-operative CBCT scans, no additional imaging procedures were necessary. Subjects agreed to the use of their existing tomographic data in clinical research. The study took place at a tertiary referral medical center with a cochlear-implant program. Positive votes from the local ethics committee (reference: D 522/21; ethics committee, medical faculty, University Kiel) and data protection office were obtained.

### Subjects

For the study, a training dataset consisting of 85 CBCT scans on adult subjects (38 female, 47 male) with a Nucleus® CI532 or CI632 Slim Modiolar electrode array was analyzed. Median age at implantation was 63 years (interquartile range: 15 years). The dataset had CBCT scans of 54 electrode arrays implanted on the right ear and 31 arrays from the left ear, whereby both sides were analyzed in one subject of a total of 27 bilateral subjects included in the study. One experienced surgeon implanted all devices. Surgeries were performed in accordance with the manufacturer’s surgical guideline and all the arrays were inserted using the round window approach. The following inclusion criteria were met: full insertion of the electrode array into the cochlea; void of tip-foldover; void of cochlear malformation; void of reinsertions of the array.

### Image pre-processing

A CBCT machine (KaVo 3D eXam) performed head scans with an isotropic voxel size ≤ 0.25 mm, and the DICOM files were pre-processed using the RadiAnt DICOM viewer (Medixant). The curvature to be measured are extracted from a two-dimensional cross-sectional plane perpendicular to the mid-modiolar axis and coplanar to the basal turn of the cochlea, i.e. ‘cochlear view’ (Fig. 1) [4, 23, 27, 28]. This section plane was manually prepared by multiplanar reconstruction for each CBCT scan, and exported in the jpeg format to be used for the curvature measurements.

### A priori categorization of electrode position

For the analysis of curvature, the electrode position was a priori categorized into three groups: regular, under-insertion and over-insertion. Electrode arrays were categorized as under-insertion if the angular depth of insertion (aDOI) at electrode contact E22 was below the 10th percentile (342°; median 385°; 90th percentile 412°). With the use of the cochlear view, the aDOI was measured relative to the chord produced between the mid-modiolar axis and the center of the round window (0°) [4, 22, 26]. An expert, with experience in evaluating the electrode placement, categorized over-inserted electrode arrays with visual inspection of the cochlear view. A categorization to the over-insertion group was indicated by a visible bending of the electrode array towards the lateral wall at medio-basal contacts (see example in Fig. 1). This is comparable to the category “poor” by subjectively rating coronal CBCT images in Aschendorff et al. [11]. An electrode position was categorized as poor if electrode contacts are closer to the lateral wall than to the inner wall of the cochlea and this typically appeared at insertion depths between 45° and 180° [11]. Regular was categorized if the criteria of under-insertion and over-insertion did not apply.

### Curvature analysis

**Fig. 2:**
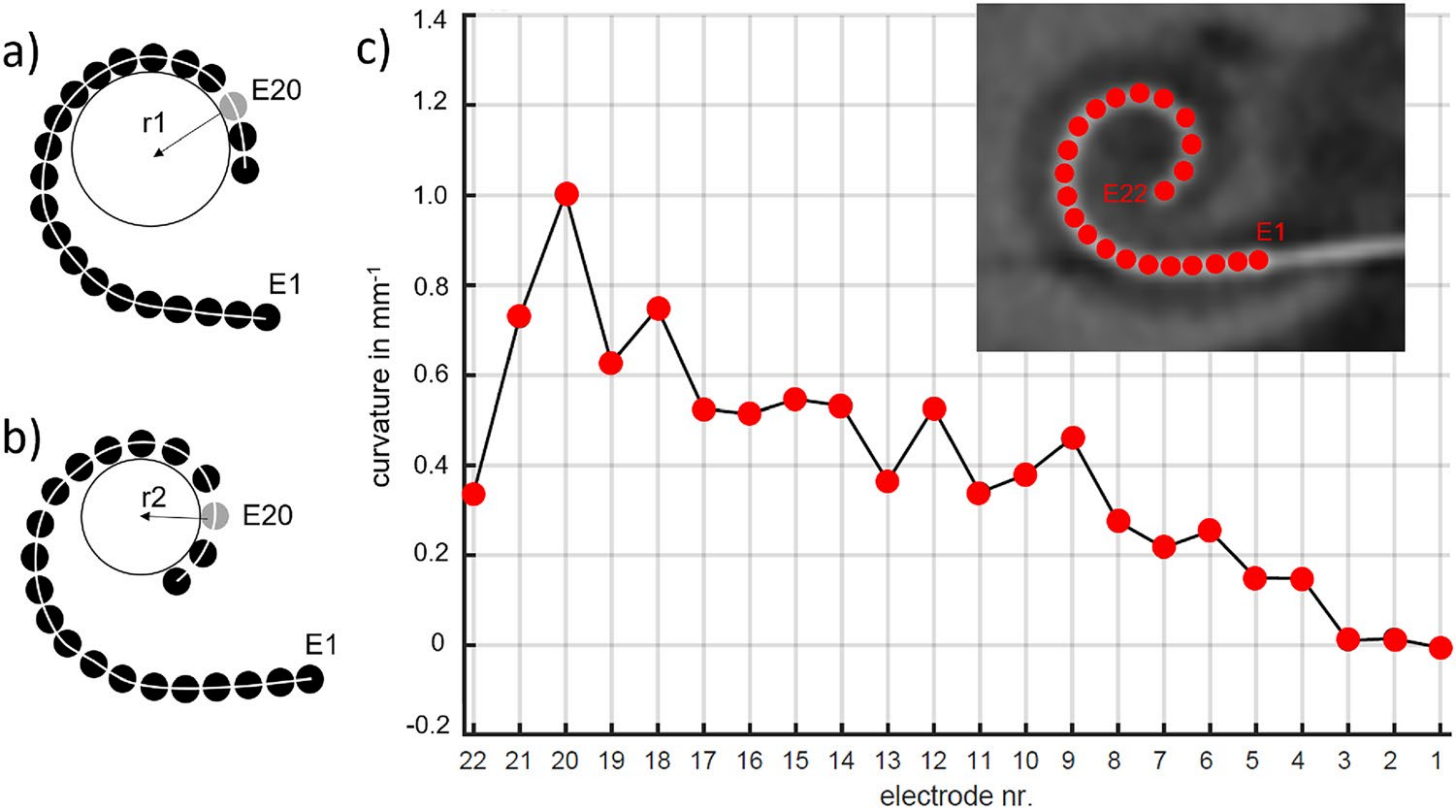
**a** and **b** Schematic of the curvature at electrode contact E20 (grey). The curvature at each electrode contact can be thought as the reciprocal of the radius r of the circle. The higher the curvature of the electrode array around E20, the smaller the radius r of the circle, and vice versa. **a** Illustration of an electrode array with low curvature at E20 and large radius r1; **b** Array with high curvature at E20 and small radius r2. The white line through the electrode contacts indicates an ideal fitted spline curve. **c** Example showing the curvature measured at all 22 contacts of an electrode array that has been categorized as regular electrode position

The open-source Fiji plugin “Kappa” was used by applying a semi-automatic procedure to measure the curvature on the basis of cubic B-splines [29]. Cubic B-splines are composed of piecewise 3rd degree Bézier curves [29], with the 22 electrode contracts as control points. To explain the curvature, given a point P on a curve and a circle that tangents the curve at this point. All other points of the curve determine the circle’s radius. The higher the curvature of the curve around point P, the smaller the radius of the circle, and vice versa. That is, the curvature at each electrode contact (point P) can be thought of as the reciprocal of the radius of the circle (Fig. 2a, b).

After importing the cochlear view into the Fiji software, the JPEG was recalibrated. In the absence of calibration information, distances in Fiji were measured in the unit pixel. By using a reference line and its known length (in mm), each JPEG was recalibrated so that the curvature measurements were comparable between the images. As the reference line, the chord produced between the mid-modiolar axis and the center of the round window was drawn by using the RadiAnt DICOM viewer and saved in each JPEG. Both, the JPEG and the scale factor (mm/pixel) were then imported into the Kappa plugin. An initialization curve was created by clicking each of the 22 electrode contacts. All electrode contacts were used as control points to automatically fit a B-spline curve by applying an iterative minimization algorithm (Fig. 2) [29, 30]. The local curvature at each of the 22 electrode contacts was derived from the fit curve and exported in the csv format for statistical analysis. Figure 2c exemplarily shows the measured curvature along of an electrode array with 22 contacts. As the measured curvature along electrode arrays had a reverse sign for right and left ears, all measurements were converted to the right ear. That is,  – 1 multiplied the measured curvature of left-sided electrode arrays.

The bowing ratio and the mean curvature were calculated from the curvature across the electrode array to categorize over-inserted arrays and those with under-insertion by using individual curvature variables. The bowing ratio was defined as the ratio of the mean curvature at contacts E14–16 to the mean curvature at E7–8 (Eq. 1).

1 $${\text{bowing ratio}} = \frac{{\overline{{{\text{curvature}}_{E14 - 16} }} }}{{\overline{{{\text{curvature}}_{E7 - 8} }} }}$$

Testing the bowing ratio and the mean curvature as classifiers, two ROC analysis were conducted to develop the optimal threshold value to categorize a group with an sub-optimal electrode position (over-insertion or under-insertion) from the regular group. The true positive rate (TPR) and false positive rate (FPR) were calculated for a range of equidistant threshold values. These values were selected by a visual inspection of the data distribution and boxplot of each variable. TPR was the rate at which the threshold value predicts an abnormal electrode position for cases that are sub-optimal, i.e. over-insertion or under-insertion. FPR was the rate at which the threshold value predicts an abnormal position for cases that are actually regular. The optimal threshold value was determined using the Youden’s index.

### Data analysis

Statistical analyses were performed using the MATLAB™ software (The MathWorks, Inc, Natick, Massachusetts). The variables to be analyzed were the curvature at each of the 22 electrode contacts as well as the angular depth of insertion (aDOI) at the intracochlear electrode tip.

Median values were calculated as well as the 10th and 90th percentiles, to visualize the curvature across the 85 CBCT scans for each of the 22 electrode contacts.

Boxplots show the distribution of the bowing ratio and mean curvature across the electrode arrays, whereby the line inside the box is the median, and the bottom and top edges of the box are the 25th and 75th quantiles, respectively. The interquartile range (IQR) was defined as the distance between the edges of the box. Outliers are indicated by values that are more than 1.5·IQR from the edges of the box. The whisker of each box corresponded to the minimum/maximum data value that is not an outlier.

The Wilcoxon test was applied to compare the central tendencies of two variable samples. Statistical significance was defined as p < 0.05 and Bonferroni-adjusted alpha was used in the case of multiple testing.

Analysis of the relationship was performed using the visual inspection of scatter diagrams and a correlation analysis with a linear fit model. The quality of each fit was evaluated by determining the adjusted R-squared. Analysis of variance (ANOVA) was used to assess the significance of a fit model.

## Results

### A priori categorization of electrode position

With the use of visual inspection of the 85 CBCT scans, 58 electrode arrays were categorized as regular positioned (68.2%), 19 as over-inserted arrays (22.4%) and 8 arrays had an under-insertion (9.4%).

### Analyzing the curvature along the electrode array

**Fig. 3:**
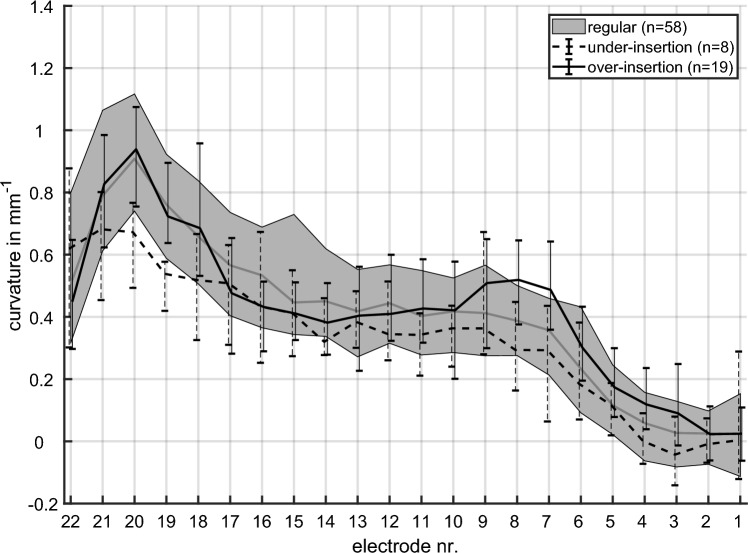
Curvature along the electrode array showing for a regular position, over-inserted electrode arrays and an under-insertion. The 10th and 90th percentiles and the median value are illustrated for each group. For the regular group, the area between these percentiles is shaded gray

Figure 3 shows the curvature along the electrode array in each of the three groups under investigation, i.e. regular electrode position, over-insertion, under-insertion. Regular electrode positions had a low curvature at basal contacts and a high curvature at apical contacts, which is associated to a pronounced bending of the electrode array. There is a peak of the regular curvature around electrode contact E20. The curvature in general increases monotonously from basal towards this peak. The curvature decreases sharply between the peak and contact E22.

Compared to the regular electrode position, there is less curvature along the electrode array with an under-insertion. That is, all electrode contacts except E22 have a median for an under-insertion that is below the median for a regular position. At contacts E19 and E20, the median for under-insertion falls below the 10th percentile of the regular group; the apical peak is thus less pronounced for the under-insertion group than for the regular group. In comparison to an under-insertion, the apical peak around E20 is not affected by an over-insertion. This is evident by the overlap of the 10th–90th percentile ranges from the regular and over-insertion group at electrode contacts E19–21. The over-insertion group rather show a secondary maximum of the curvature at E7 and E8 compared to the regular group, as the curvature of over-inserted arrays exceeds the 90th percentile of the regular group. From this peak, the curvature falls slightly towards apical before achieving a peak around contact E20.

### Categorization of under-inserted electrode arrays

**Fig. 4:**
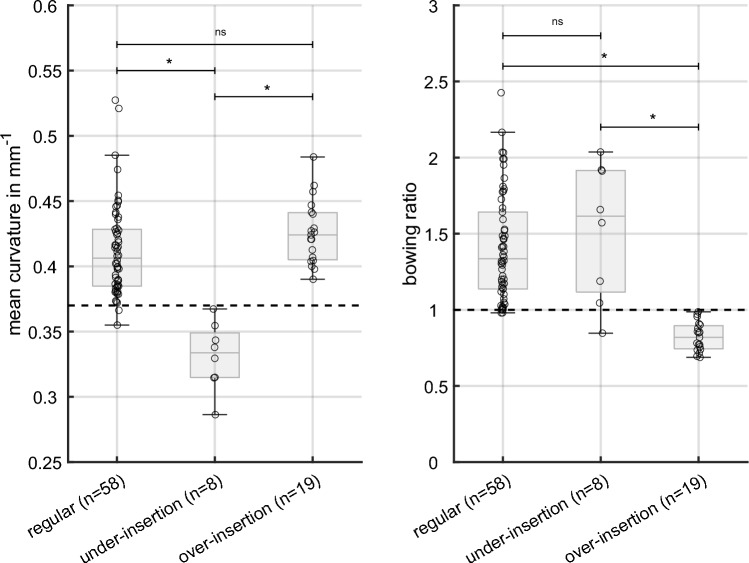
**Left panel** Mean curvature across the electrode array for a regular electrode position, position with under-insertion and position with an over-insertion. The Wilcoxon test revealed significant differences with the under-insertion group compared to the regular and over-insertion group, respectively (*p* < 0.001). The black dashed line show the optimal threshold value from ROC analysis to categorize electrode positions with under-insertion (< 342°) and normal insertion depth. **Right panel** Bowing ratio calculated from the curvature across the electrode array (Eq. 1). Results showing for electrode arrays with a regular position, position with under-insertion and position with an over-insertion. The Wilcoxon test revealed significant differences with the over-insertion group compared to the regular and under-insertion group, respectively (p < 0.001). The black dashed line show the optimal threshold value from ROC analysis to categorize electrode positions with with/without an over-insertion

The mean curvature across the 22 electrode contacts was calculated to distinguish electrode arrays with under-insertion and those with regular position. The left panel in Fig. 4 shows the mean curvature for the three groups, i.e. regular position, under-insertion and over-insertion. The mean curvature was significantly lower with an under-insertion than with regular position and an over-inserted array, respectively (p < 0.001). ROC analysis was conducted to find the optimal threshold value to categorize the group with under-inserted arrays and those with a normal insertion depth (≥ 342°). Therefore, the regular group was pooled with the over-insertion group, as no statistically significant differences were found between these two groups (p > 0.05). The true positive rate (TPR) and false positive rate (FPR) were calculated for threshold values ranged from 0.25 to 0.5 mm^−1^ with a step size of 0.01 mm^−1^. Calculation of Youden’s index revealed a value of 0.37 mm^−1^ as the optimal threshold value to categorize electrode arrays with under-insertion against those with normal insertion depth. For the optimal threshold value, TPR was 1.00 and FPR was 0.03.

### Relationship between the angular depth of insertion and the mean curvature

**Fig. 5:**
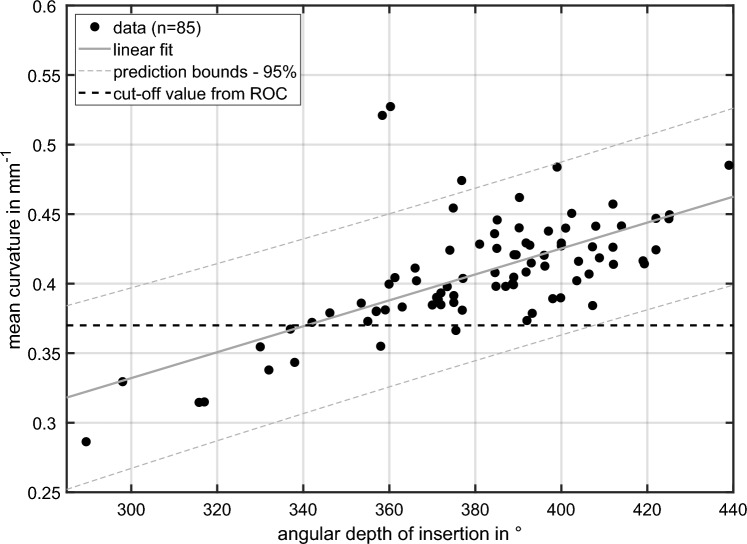
Relationship between the angular depth of insertion and the mean curvature across the electrode array for all investigated CI systems. The black dashed line show the optimal threshold value from ROC analysis to categorize electrode positions with under-insertion (< 342°) and normal insertion depth

The relationship between the angular depth of insertion and the mean curvature is shown in Fig. 5. The mean curvature increases linearly as a function of the insertion depth (p < 0.05, ANOVA). Adjusted R-squared, used to quantify the strength of the relationship, was calculated with 0.42. By applying the optimal threshold value from the ROC analysis, 10 out of 85 mean values are below 0.37 mm^−1^. Of these 10 cases, 8 had an under-insertion (< 342°) and 2 had a normal insertion depth (358° and 375°).

### Categorization of over-inserted electrode arrays

The bowing ratio was calculated from the curvature across the electrode array to categorize over-inserted arrays. Note that the over-insertion group show a second local maximum of the curvature at electrode contacts E7 and E8 compared to the regular group, and a slightly decrease of curvature from this peak towards apical before achieving the primary peak around E20 (Fig. 3). However, regular electrode positions show a monotonously increase of curvature from basal towards the peak around E20. Consequently, the bowing ratio was defined as the ratio between the mean curvature across contacts E14–16 and the mean curvature at E7–8 (Eq. 1). With a regular increase in curvature from basal to apical, the bowing ratio is > 1, and < 1 with a secondary maximum of curvature at contacts E7/E8.

The right panel in Fig. 4 shows the results of the bowing ratio for the three groups. There are significant differences in the bowing ratio for the over-insertion group compared to the regular and under-insertion group, respectively (p < 0.001). To find the optimal threshold value for categorizing electrode positions with/without an over-insertion, a ROC analysis was performed testing threshold values between 0.75 and 1.25 (step size 0.05). Youden’s index confirmed the presumption that a ratio of 1.00 is the optimal threshold value for distinguishing electrode positions with an over-insertion from those without an over-insertion. Using a bowing ratio of 1.00, the TPR was 1.00 and the FPR was 0.06. That is, all cases from the over-insertion group had a bowing ratio < 1.00, whereas 4 out of 66 cases (sum of all cases from the regular and under-insertion group) were misidentified as an over-inserted position.

## Discussion

### On the clinical relevance regarding a quantitative assessment of the CI electrode position

Awareness of the intracochlear CI electrode position is decisive in the treatment with a cochlear implant. During the operation, the surgeon needs to know if the electrode array is positioned in the cochlear as intended. Moreover, even before the initial audio processor fitting, clinicians require information on whether electrode contacts are located outside the cochlea and whether there is a kinking or tip-foldover of the electrode array. Variations in the quality of the position can occur even if the electrode array is inserted fully and without folding or kinking. This work reports results on the electrode position for a sample of the perimodiolar Nucleus® Slim Modiolar electrode. Though all electrode arrays have been implanted in accordance with the surgical guideline, in one third of all cases (31.8%) a sub-optimal position was found. These results are similar to Aschendorff et al. (2017), finding a “poor” electrode position in about 35% of cases when inserting the array to the second marker at cochlear opening [11]. Electrode-to-modiolus distances can be increased partially with both an over-insertion and under-insertion of the electrode array. Both psychoacoustic and electrophysiological thresholds in cochlear implants depend on electrode-modiolus distances [2, 4, 5, 9, 10, 21, 31–33]. Thus, information on the proximity of electrode contacts to the modiolus may be valuable if the patient’s hearing performance is less than expected or if ECAP thresholds or comfort and threshold levels are pathologically heightened. Evaluating the CI electrode position is therefore clinical relevant in individual cases. This assessment should be quantitative to ensure that the test results are standardized and therefore comparable. Subjective ratings, conversely, have the risk of diagnostic errors due to a combination of system-related factors and cognitive-perceptual biases in both experienced and inexperienced raters [26, 34–38].

### Advantages and limitations of curvature analysis

There are various methods to measure electrode-to-modiolus distances and the insertion depth, both manually and automatically. However, there is a large variability in the results due to challenge of detecting anatomical landmarks such as the round window and the mid-modiolar axis [25, 26]. In contrast, electrode contacts can be identified robustly due to the low beam transparency relative to the surrounding tissue. Thus, an analysis of the curvature at the electrode contacts can be used as a basis for evaluating the intracochlear electrode position. Measuring the curvature with the Kappa plugin in the open-source software Fiji is semi-automatic, i.e. users simply have to mark the intracochlear electrode contacts and the software then calculates the curvature at each electrode contact automatically. These qualities offer the potential to apply the method to modified Stenver’s view X-ray where there are no identifiable landmarks other than the electrode contacts.

It was shown in this work that by simplifying the curvature along the array down to single quantities (mean curvature, bowing ratio), both over-inserted and under-inserted arrays can be detected with high true positive rate and low false positive rate. Despite the advantages of curvature analysis over clinically established electrode localization, there are also some limitations. There are concerns of measurement inaccuracies due to the fit of the B-spline to the true shape of the electrode array using the cochlear view, as well as due to this plane itself. The cochlear view is a sectional plane to be coplanar with the basal turn of the cochlea. This plane was reconstructed manually by the three-dimensional volume data set and was therefore vulnerable to system-related errors and cognitive-perceptual biases.

### Relationship between the angular depth of insertion and the curvature

The insertion depth measured at the tip of the CI electrode array can be of interest for clinical issues (anatomy-based fitting [39–43]) as well as for the development of new electrode arrays. The precise insertion depth at the most apical electrode contact is of minor relevance with the Slim Modiolar electrode that was investigated here. According to the physician’s guide, a full insertion of the Slim Modiolar is performed if one of the three markers are at the cochleostomy or round window opening. For this type of electrode, Aschendorff et al. found that the angular depth of insertion at contact E22 did not differ significantly depending on the insertion marker at cochlear opening [11]. Thus, using the physician’s guide, the tip of the Slim Modiolar electrode will be located within a range of insertion depth that is affected by the cochlear size and shape. With the curvature analysis, we present a simple tool to describe whether this electrode type is inserted deep enough or not. This can be analyzed using the mean curvature across the electrode array, as it differs significantly between an insertion depth below and above 342°. Note that this threshold on insertion depth has been defined on statistical rationale, i.e. defining the 10th percentile of aDOI as cut-off to categorize under-inserted electrode arrays. In addition, the group with an insertion depth ≥ 342° showed an apical peak around electrode contact E20, that is associated with the strong curvature of the array around approx. 360° insertion depth. If the array was not inserted deep enough (< 342°) the peak around E20 was less pronounced. Thus, even though a significant relationship was found between the mean curvature and the angular depth of insertion, curvature cannot be used to predict the aDOI accurately. This is due to the variability of the data (Fig. 5).

### Methodical limitations

The aim of the present study was to investigate if an analysis of the curvature in perimodiolar electrode arrays can be used to assess the quality of the intracochlear position. For this, CBCT scans of a training data set were analyzed and categorized into three groups, i.e. regular position, over-insertion, under-insertion. This categorization was based on a subjective rating according Aschendorff et al. [11] and Banalagay et al. [16] as well as the data distribution. The methods used for categorization are not a gold standard that are calibrated to be highly accurate. That is, the results of the curvature analysis were merely related to a clinically established method. Other raters may have come to a different subjective view of the electrode’s position. All the electrode arrays having been implanted by a single surgeon also limit the objectivity of the curvature data.

Part of the aim of this study was to find individual curvature variables and to develop optimal threshold values for each of them to categorize electrode arrays with under-insertion and over-insertion. For this reason, the optimal threshold value to separate the categories was determined for each variable using an ROC analysis. The ROC analysis was therefore used here to establish the threshold values, but not to validate the results. It is necessary to validate the algorithms using additional data sets to avoid overestimating the performance of the developed threshold values.

## Conclusions

Perimodiolar electrode arrays have been designed to yield low stimulation thresholds and a focused stimulation by a close position to the modiolar wall. The intracochlear placement of perimodiolar electrode arrays may result in a sub-optimal position, even if the surgical guideline is adhered. With the Nucleus® Slim Modiolar electrode array investigated here, over-insertion of the array occurred in 22.4% of cases and 9.4% of the arrays had an under-insertion in the cochlea in the investigated population. Such sub-optimal array positions are due to the large variability in cochlear size and shape, affecting the interface between the electrode contacts and spiral ganglion neurons. In clinical practice, the electrode position is assessed either by subjective rating or by measuring the electrode position relative to landmarks of the cochlea. These methods are limited as being either not standardized (subjective rating) or dependent on the difficulties in identifying anatomical landmarks (electrode-to-modiolus distance, angular depth of insertion). In this work, we present an approach to evaluate the quality of the electrode position based on a curvature analysis of the intracochlear electrode array. This method is quantitative, semi-automatic and independent of critical anatomical landmarks such as the round window and mid-modiolar axis. The curvature analysis can be used to assess if a CI electrode array has been inserted deep enough into the cochlea, but curvature cannot be used to predict the angular depth of array insertion accurately. It was shown that by deriving the curvature at the electrode contacts from the CBCT image to simple quantities, both cases of over-inserted arrays and under-inserted arrays could be categorized with a high true positive rate and low false positive rate. These results must be validated using additional data sets. Reference data should be collected by other centers and other surgeons. This applies also on other electrode types that differ in size and shape from the electrode arrays investigated here. Furthermore, it is of clinical interest to investigate whether the electrode position categorized by curvature analysis has an influence on the patients performance with CI.

## Data Availability

Data is available on reasonable request.
